# The Role of Intrasexual Competition and the Big 5 in the Perpetration of Digital Dating Abuse

**DOI:** 10.1177/14747049241288188

**Published:** 2024-10-09

**Authors:** Manpal Singh Bhogal, Morgan Taylor

**Affiliations:** School of Psychology, 8695 University of Wolverhampton, Wolverhampton, UK

**Keywords:** digital dating abuse, mate retention, big five, intrasexual competition, agreeableness

## Abstract

Recent research has examined digital dating abuse through an evolutionary lens, finding people who report intrasexual competitiveness engage in digital dating abuse. Here, we replicated this finding and extended the literature by examining the role of the Big Five personality traits in the perpetration of digital dating abuse, which, to our knowledge, has not been examined in relation to digital dating abuse (*n*=280). This paper reports findings showing intrasexual competitiveness positively predicts the perpetration of digitaldating abuse; whereby high intrasexual competition is related to high levels of digital dating abuse. Agreeableness was a negative predictor of digital dating abuse; whereby high agreeableness was related to low perpetration of digital dating abuse. Our findings extend the literature exploring digital dating abuse through an evolutionary lens.

Intimate partner violence (IPV) is a public health concern. A recent form of IPV is digital dating abuse, which involves the use of technology to perpetrate electronic intrusive behaviors towards a romantic partner (Reed et al., 2016). These behaviors can involve monitoring a partners’ location, their ‘last seen’ on instant messaging apps, controlling who they engage with online and isolating their partner through manipulation and control (Zweig et al., 2013).

Previous research has examined digital dating abuse through an evolutionary lens, suggesting people engage in digital dating abuse using information communication technologies with an aim to deter romantic rivals from an existing relationship (Atari et al., 2017; Bhogal & Howman, 2019; Bhogal et al., 2019; Bhogal et al., 2022; Bhogal & Wallace, 2022; Brem et al., 2015). Intrasexual competition refers to rivalry displayed towards same-sex romantic competitors, with an aim to increase reproductive success (Miner et al., 2009). This can be in the form of rival derogation and self-promotion (see Polo et al., 2018). Cyber dating abuse allows one to gauge cues of infidelityrelated to potential romantic partners, thus deterring romantic rivals, increasing ones reproductive success. In support, recent research has shown that there is a positive relationship between intrasexual rivalry and digital dating abuse perpetration (Bhogal et al., 2022).

Research shows strong links between romantic jealousy and the perpetration of digital dating abuse, in that those who experience jealousy, engage in digital dating abuse (Branson & March, 2021). As jealousy is often heightened with intrasexual rivalry, those who report greater intrasexual competition should perpetrate digital dating abuse, which has only recently been explored by Bhogal et al. (2022). They argue that intrasexual competition can deter romantic rivals/poachers and keep a relationshop intact through monitoring a partners online activities. In support, those who engage in intrasexual competition fear being replaced by higher quality romantic rivals (Miner et al. 2009). Cyber dating abuse therefore, could prevent partner switching through online monitoring. Consistent with Bhogal et al. (2022), we used an intrasexual competition scale which measures competition as well as rival derogation.

As research shows that men and women experience intrasexual rivalry differently (Keys & Bhogal, 2018), we examined the role of gender in digital dating abuse perpetration. However, the evidence relating to digital dating abuse and gender is inconclusive. For example, some studies show that men perpetrate to a greater degree than women (Deans & Bhogal, 2019; Smith et al., 2018), some show women perpetrate more than men (Zweig et al., 2013), and some studies show men and women report similar rates of perpetration (Bhogal et al., 2019; Bhogal et al., 2022). A recent meta-analysis found that most studies in the area show that men and women perpetrate digital dating abuse at similar rates (Martinez-Soto & Ibabe, 2024).

Researchers have argued that to better understand digital dating abuse, scholars should explore the determinants that drive this behaviour (Plouffe et al., 2022). The Big Five personality traits consist of conscientiousness, openness, extraversion, neuroticism and agreeableness. Although the Big Five personality traits have been found to be strongly related to offline IPV (see Ulloa et al., 2016) and aggressive behaviours in romantic relationships (Hines & Saudino, 2008), no research (to our knowledge) has explored the role of the big 5 in online dating abuse.

The Big Five provide a useful framework for understanding IPV, and in this instance, digital dating abuse. For example, individuals high in neuroticism engage in maladaptive coping strategies, which can include hostile and impulsive behaviors (Dam et al., 2021) concurrent with digital dating abuse. Further, low levels of agreeableness are associated with hostility, and a lack of empathy, leading to behaviors aimed at punishing others (Ulloa et al., 2016). Agreeableness shows an inverse relationship with interpersonal aggression suggesting individuals low in agreeableness are more prone to engaging in hostile behaviors (Barlett & Anderson, 2012; Tremblay & Ewart, 2005). Further, neuroticism has been linked to aggressive behaviours towards partners, indicating that individuals high in neuroticism may respond defensively or emotionally in interpersonal conflicts (Barlett & Anderson, 2012; Egan & Lewis, 2011).

Further, in the context of cyberbullying, higher scores on extraversion and lower scores on agreeableness and conscientiousness are associated with increased perpetration of abusive behaviours in relationships (Festl & Quandt, 2013). Low agreeableness and conscientiousness could contribute to behaviors aimed at controlling or manipulating partners online (Festl & Quandt, 2013), behaviours concurrent with digital dating abuse. Despite these insights, research on digital dating abuse and personality is limited. Van de Weijer and Leukfeldt (2017) argue for further exploration to understand better how personality traits shape interactions in online relationships and contribute to the perpetration of digital dating abuse.

These findings illustrate how individual differences in personality traits can shape behaviours within romantic relationships, and here, we applied these findings to further exploring determinants of digital dating abuse. Due to the importance of replication in psychological science, here, we replicated the findings of Bhogal et al. (2022) who found that intrasexual competitiveness positively predicts digital dating abuse perpetration.

There were two aims of this study 1) to explore whether the big 5 personality traits were associated with digital dating abuse perpetration and 2) to replicate previous work on whether intrasexual competition is related to digital dating abuse perpetration.

Hypotheses:

^1^Exploratory: Extraversion, conscientiousness, openness, agreeableness, and neuroticism would be related to the perpetration of digital dating abuse.

Hypothesis 1:
Intrasexual competition would be positively related to the perpetration of digital dating abuse.

## Method

### Design and Participants

We adopted a cross-sectional correlational design to explore whether the Big Five personality traits (openness, neuroticism, agreeableness, extraversion, and conscientiousness), intrasexual competition, and sex were associated with digital dating abuse perpetration.

Two-hundred eighty heterosexual participants took part (44 men, 236 women, mean age=23.6, *SD*=6.18) who were in a romantic relationship at the time of participation (mean relationship length=78.91 months, *SD*=82.61). Participants were recruited via social media sites and the department's research participation scheme (the sample included members of the public and students).

To guide our anticipated sample size, an a-priori power analysis was conducted using G*power. To achieve 80% power (effect size of 0.13 – comparable to Bhogal et al. 2022, with 7 predictors and an alpha level of 0.05) G*power recommended 118 participants which was surpassed.

### Procedure

Once providing informed consent, participants were asked to complete a demographics section asking their sex, relationship length, and age. Following this, participants were asked to complete scales mentioned in the material section. Lastly, participants were fully debriefed online. Data were collected anonymously online through Qualtrics, which is an online survey builder.

## Materials

### Intrasexual Rivalry Scale (Karimi et al., 2019)

The intrasexual rivalry scale is a 16 item, self-report measure measuring intrasexual rivalry/competition (measured on a 4-point Likert scale, 1= not at all applicable, 4= completely applicable). The scale includes male pronouns which were altered depending on the sex of the participant. The female version was presented to women and the male version was presented to men. The questions remained the same, with altered gendered pronouns. For example, “*I look for negative points in attractive women*” was presented to women and “*I look for negative points in attractive men*” was presented to men. Cronbach's alpha values show that this scale was reliable in our sample (α= 0.85, Men, α= 0.87, Women α= 0.83).

### Digital Dating Abuse Scale (Reed et al., 2016)

The 19-item digital dating abuse scale was used to measure participants’ engagement in digital dating abuse perpetration. Participants were asked to score each item based on how often they engaged in the behaviours during the past 12 months. Questions were answered on a 3-point Likert scale (“0= never, 3= very often”). Example items include “*Looked at my partners private information on a computer or mobile phone without permission”* and *“Pretended to be my partner on a mobile phone or the internet without permission”.* This scale was reliable in our sample (α=.74).

### The Big 5 Inventory (BFI) (Goldberg, 1993)

The Big 5 inventory is a 44-item scale measuring the Big Five personality traits (extraversion, agreeableness, neuroticism, openness, conscientiousness). The scale includes items to measure each personality trait. Questions were answered on a 5-point Likert scale (“1= disagree strongly, 5= agree strongly”). An example item for agreeableness is “*Is helpful and unselfish with others*”; for extraversion “*Is talkative*”; for conscientiousness “*Does a thorough job*”; for neuroticism “*Can be tense*” and for openness “*Is original, comes up with new ideas*”. Reliability analyses show that the sub-scales were reliable in our sample; extraversion (α=.82), agreeableness (α=.69), conscientiousness (α= .74), neuroticism (α=.84) and openness (α=.68).

## Results

Descriptive statistics are displayed in Table 1. Bivariate Pearson's correlations were conducted to examine relationships between all predictors, which are presented in Table 2. Regression values for all key variables are displayed in Table 3.

**Table 1.** Descriptive Statistics Relating to all key Variables (Totals Calculated for Each variable).

Variable	Total (*SD*)
Extraversion	25.68 (6.05)
Agreeableness	34.61 (5.13)
Conscientiousness	33.03 (5.23)
Neuroticism	27.07 (6.44)
Openness	35.26 (5.41)
Intrasexual competition	30.93 (7.36)
Digital dating abuse	21.08 (2.33)

**Table 2.** Bivariate Correlations Between key Variables.

Variable	1	2	3	4	5	6	7
1. Extraversion	__						
2. Agreeableness	.058	__					
3. Conscientiousness	.134*	.224**	__				
4. Neuroticism	−.423**	−.151*	−.210**	__			
5. Openness	.277**	.093	.070	−.213**	__		
6. Intrasexual competition	.159**	−.193**	−.001	.027	.126*	__	
7. Digital dating abuse	.020	−.200**	−.119*	.121*	−0.19	.223**	__

**p < .01. *p < 0.05.

**Table 3.** Results of the Multiple Regression Model.

Variable	β	*P*	*t*	CI (95%) Lower	Upper	Tolerance	VIF
Sex	.064	.326	.984	–407	1.221	.794	1.260
Extraversion	.050	.447	.761	–031	.070	.757	1.322
Agreeableness	–141	.022	−2.297	–119	-.009	.881	1.135
Conscientiousness	–079	.193	−1.305	–088	.018	.909	1.100
Neuroticism	.079	.256	1.138	–021	.077	.698	1.432
Openness	–011	.855	-.182	–057	.047	.881	1.135
Intrasexual competition	.206	.001	3.268	.026	.104	.842	1.188

A small positive correlation was found between extraversion and intrasexual competition suggesting those who are more extraverted engage in more intrasexual rivalry. A small negative correlation was found between agreeableness and intrasexual competition, suggesting those who are more agreeable, engage less in intrasexual rivalry. A small to moderate negative correlation was found between agreeableness and digital dating abuse, suggesting those who are more agreeable engage less in digital dating abuse. A small negative correlation was found between conscientiousness and digital dating abuse suggesting those who are higher in conscientiousness engage less in digital dating abuse. A small positive correlation was found between neuroticism and digital dating abuse suggesting those who are more neurotic engage in more digital dating abuse. A small positive correlation was found between openness and intrasexual rivalry suggesting those who are more open engage in more intrasexual competition. A small to moderate positive correlation was found between intrasexual competition and digital dating abuse suggesting those who score high in intrasexual rivalry also engage more in digital dating abuse.

Multiple regression was conducted to examine whether the participant's sex, intrasexual competition, openness, conscientiousness, agreeableness, neuroticism, and extraversion were associated with the perpetration of digital dating abuse (see Table 3 for statistics from the regression analysis). Preliminary analyses show that there was no violation of the assumption of independent errors and multicollinearity.

All variables were entered into the model simultaneously. The model was statistically significant (*F* (7, 272) =4.048, *p* <.001; *r*=.307, Cohens *f2*=.44) explaining 30.7% of the variance in digital dating abuse perpetration. Intrasexual competition was a positive, significant predictor of digital dating abuse, showing that high intrasexual competition explained the perpetration of digital dating abuse. Agreeableness was a negative, significant predictor of digital dating abuse, showing that high agreeableness explained low engagement in the perpetration of digital dating abuse. All other predictors were non-significant in the model.

## Discussion

Our findings show that those who scored higher in agreeableness engaged less in the perpetration of digital dating abuse, which is consistent with previous work on agreeableness, cyberbullying and interpersonal aggression outlined in the introduction. There were significant correlations amongst the Big Five and digital dating abuse, however only agreeableness was a significant predictor in the regression model.

Those who engaged more in intrasexual competition also engaged more in digital dating abuse supporting hypothesis 2, thus replicating previous work (Bhogal et al., 2022). Agreeableness was a significant predictor of digital dating abuse perpetration in that high agreeableness was associated with low engagement in digital dating abuse. This is a novel finding as the Big Five have not previously been explored in relation to digital dating abuse perpetration. This finding is somewhat consistent with previous work showing agreeableness to have a negative association with, cost-inflicting behaviours in relationships, in that those who were high in agreeableness engaged less with cost-inflicting mate retention behaviours (Kardum et al., 2020) and low agreeableness scores are related to an increase in cost inflicting mate retention behaviours such as digital dating abuse (Miguel & Buss, 2011). This finding adds modest support to the role of personality factors in the perpetration of digital dating abuse. Further empirical investigation is needed in examining the role of the Big Five personality traits in the perpetration of digital dating abuse.

In the interest of conceptual replication, and considering its important in psychological science (Fetterman & Sassenberg, 2015), we successfully replicated recent findings suggesting intrasexual competition explains the perpetration of digital dating abuse (Bhogal et al., 2022). Such replications are important in advancing the state of psychological science and adding trust to existing research findings (Nosek et al., 2012). Furthermore, Van Ouytsel et al. (2020) argue the need for researchers to adopt a variety of theoretical frameworks to better understand and prevent digital dating abuse perpetration.

The fact that there was a gender imbalance in our sample is a limitation. Future work should aim to recruit an equal sample of men and women, as our findings cannot necessarily be generalised to both men and women, considering most of the sample were women. Future research should also examine factors associated with victimization and perpetration in digital dating abuse.

There are policy implications of this work. Education programs in schools and colleges could benefit from our work by incorporating the findings into educational programmes regarding healthy digital behaviour amongst romantic couples, and the role of personality and competitive behaviors.

Quiroz et al. (2024) argue the importance of continually examining cyber dating abuse in young people, particularly as young people differ from older people in that they have integrated their online and offline worlds. Therefore, the online world to young people is as real as the offline world. Therefore, it is surprising that most interventions related to dating violence predominantly focus on offline violence when many interactions, if not most interactions amongst young adults take place online (Galende et al., 2020). This should be a focus for future research.

Interventions for dating violence have focused on providing support to female victims of cyber dating as opposed to male victims (Martinez-Soto & Ibabe, 2024). This does no favours to female perpetrators who seek interventions to support and better understand their online behaviour. Interventions should focus on supporting people regardless of gender in their understanding of directing abusive behaviours in romantic relationships. Furthermore, most research into cyber dating abuse rarely focuses on cyber dating abuse and has instead tailored interventions on cyber dating abuse to focus more on offline abuse (Li et al., 2023). The lack of interventions and practical solutions to preventing cyber dating abuse could be adding to its increased prevalence. To our knowledge, one systematic review has been conducted on the effectiveness of interventions to prevent cyber dating abuse, which only included 4 studies, showing the lack of interventions designed to prevent digital dating abuse (Galende et al., 2020). There is danger that although research into digital dating abuse is increasing, the issue is not being addressed in policy thus leading to inaction (Ramirez-Carrasco et al. 2023).

Raising awareness is of critical importance in tackling digital dating abuse. With the rise of technological use in adolescence, educational resources in schools should educate people on the dangerous uses of technology such as password sharing, private information, and the factors that can be related to cyber dating abuse, thus providing people with knowledge of the signs of dating violence (Martinez-Soto & Ibabe, 2024).

### Limitations

Although there are strengths to this piece of research, there are notable limitations. First, we did not examine cultural diversity in relation the variables being explored. The aim of this work was to explore cyber dating abuse in the UK; however, the UK is multicultural and diverse in nature. Therefore, our findings can only be applied to WEIRD samples (Western, Educated, Indistrialused, Rich and Democratic – see Henrich et al., 2010). Second, our findings are correlational in nature, therefore cause and effect cannot be inferred. Thirdly, and consistent with Bhogal et al. (2022) our reporting of cyber dating abuse relies on retrospective, self-report data which may not be as accurate as research using journals or longitudinal designs.

## Notes

1. No direction has been stated due to the exploratory nature of examining the relationship between the Big 5 and digital dating abuse.
